# Supplementary material for: Identification of HIV-reservoir cells with reduced susceptibility to antibody-dependent immune response
Source: eLife. 2022 May 26;11:e78294. doi: 10.7554/eLife.78294 (PMC9177146; doi:10.7554/eLife.78294)
Supplement: Supplementary file 1. [file elife-78294-supp1.docx]

**Supplementary File 1.** Clinical data of PLWH included in the study.

| # Patient ID | Time since HIV diagnosis (months) | CD4 Cell Count (cells/µl) | %CD4 | Viral Load (copies/ml) | Time on ART- VL-suppressed (months) | ART regimen |
| --- | --- | --- | --- | --- | --- | --- |
| 1 (EC) | 287 | 1170 | 52.8 | <50 | - | UNT |
| 2 (EC) | 11 | 840 | 23.7 | <50 | - | UNT |
| 3 (EC) | 51 | 1030 | 36.0 | <50 | - | UNT |
| 4 (EC) | 104 | 1610 | 46.0 | <50 | - | UNT |
| 5 (EC) | 59 | 890 | NA | <50 | - | UNT |
| 6 (EC) | 321 | 490 | 20.4 | <50 | - | UNT |
| 7 (EC) | 189 | 410 | 23.2 | <50 | - | UNT |
| 8 | 221 | 1240 | 40.1 | <50 | 158 | EFV+TDF+3TC |
| 9 | 373 | 960 | 33.7 | <50 | 113 | COB+DRV |
| 10 | 301 | 1050 | 27.0 | <50 | 142 | COB+DRV |
| 11 | 102 | 1040 | 37.0 | <50 | 61 | DRV+RTV+3TC |
| 12 | 128 | 760 | 28.1 | <50 | 59 | 3TC+ABV+RPV |
| 13 | 73 | 660 | 40.4 | <50 | 67 | 3TC+ABV+NVP |
| 14 | 333 | 670 | 24.1 | <50 | 86 | RPV+COB+DRV |
| 15 | 67 | 830 | 28.6 | <50 | 62 | 3TC+ABV+RPV |
| 16 | 142 | 630 | 36.0 | <50 | 85 | FTC+RPV+TDF |
| 17 | 213 | 1000 | 42.4 | <50 | 76 | FTC+RPV+TDF |
| 18 | 107 | 600 | 31.0 | <50 | 105 | FTC+RPV+TDF |
| 19 | 161 | 1150 | 36.5 | <50 | 85 | FTC+NVP+TDF |
| 20 | 90 | 710 | 38.3 | <50 | 85 | FTC+TDF+COB+ATV |
| 21 | 136 | 810 | 44.3 | <50 | 90 | 3TC+ABV+EFV |
| 22 | 86 | 1000 | 37.0 | <50 | 66 | FTC+RPV+TDF |
| 23 | 319 | 689 | 24.5 | <50 | 115 | ETV+RTG+RTV+DRV |
| 24 | 54 | 530 | 24.0 | <50 | 51 | RTG+3TC+ABV |
| 25 | 119 | 750 | 27.9 | <50 | 97 | 3TC+ABV+DTG |
| 26 | 95 | 660 | 49.5 | <50 | 69 | ETV+FTC++TDF |
| 27 | 323 | 2090 | 46.0 | <50 | 43 | 3TC+ABV+DTG |
| 28 | 240 | NA | 25.6 | <50 | 71 | TDF+COB+DRV |
| 29 | 76 | 540 | 25.0 | <50 | 56 | RPV+FTC+TDF |
| 30 | 65 | 570 | 26.2 | <50 | 62 | 3TC+ABV+RTG |
| 31 | 248 | 1050 | 32.0 | <50 | 84 | COB+DRV |
| 32 | 293 | 560 | 24.4 | <50 | 80 | RTG+COB+DRV |
| 33 | 113 | 600 | 38.7 | <50 | 95 | EFV+FTC+TDF |
| 34 | 267 | 570 | 18.1 | <50 | 98 | RTG+FTC+TAF |
| 35 | 134 | 870 | 48.7 | <50 | 97 | COB+DRV+DTG |
| 36 | 98 | 680 | 54.0 | <50 | 86 | 3TC+ABV+RPV |
| 37 | 54 | 350 | 24.0 | <50 | 48 | RTV+FTC+TDF |
| 38 | 121 | 850 | 35.6 | <50 | 88 | 3TC+ABV+DTG |
| 39 | 286 | 1060 | 30.3 | <50 | 82 | 3TC+ABV+DTG |
| 40 | 99 | 930 | 46.0 | <50 | 79 | RPV+FTC+TDF |
| 41 | 94 | 1160 | 31.0 | <50 | 73 | 3TC+ABV+DTG |
| 42 | 73 | 310 | 13.3 | <50 | 67 | 3TC+ABV+RPV |
| 43 | 99 | 450 | 25.0 | <50 | 96 | 3TC+ABV+RPV |
| 44 | 89 | 690 | 35.2 | <50 | 85 | RPV+FTC+TDF |
| 45 | 98 | 800 | 40.2 | <50 | 89 | FTC+RTV+TAF |
| 46 | 63 | 660 | 29.8 | <50 | 24 | BIC+FTC+TAF |
| 47 | 116 | 550 | 38.4 | <50 | 92 | FTC+RPV+TDF |
| 48 | 169 | 1040 | 37.4 | <50 | 101 | 3TC+ABV+NVP |
| 49 | 146 | 860 | 37.4 | <50 | 54 | RPV+FTC+TDF |
| 50 | 60 | 890 | 34.5 | <50 | 55 | 3TC+ABV+DTG |
| 51 | 40 | 570 | 27.4 | <50 | 28 | 3TC+ABV+DTG |
| 52 | 214 | 890 | 37.0 | <50 | 99 | EFV+FTC+TDF |
| 53 | 135 | 790 | 27.5 | <50 | 90 | RPV+FTC+TDF |
| 54 | 90 | 640 | 24.2 | <50 | 62 | DRV+FTC+TDF+RTV |
| 55 | 193 | 890 | 34.1 | <50 | 85 | EFV+FTC+TDF |
| 56 | 80 | 500 | 28.6 | <50 | 72 | RPV+FTC+TDF |
| 57 | 178 | 850 | 39.5 | <50 | 81 | EFV+FTC+TDF |
| 58 | 103 | 1170 | 44.0 | <50 | 58 | RPV+FTC+TDF |
| 59 | 72 | 800 | 23.1 | <50 | 18 | ABV/3TC+DTG |
| 60 | 33 | 490 | 21.0 | <50 | 28 | TDF/FTC+EVG/c |
| 61 | 31 | 560 | 27.9 | <50 | 11 | TDF/FTC+EVG/c |
| 62 | 49 | 1070 | 38.1 | <50 | 39 | TDF+FTC+EFV |
| 63 | 31 | 810 | 34.7 | <50 | 22 | ABV/3TC+DTG |
| 64 | 42 | 1150 | 37.0 | <50 | 37 | ABV/3TC+RPV |
| 65 | 13 | 1760 | 57.7 | <50 | >13 | TDF/FTC+EVG/c |
| 66 | 55 | 970 | NA | <50 | 45 | ABV/3TC+DGT |
| 67 | 168 | 540 | NA | <50 | 54 | TDF/FTC+EVG/c |
| 68 | 38 | 920 | 39.8 | <50 | 27 | COB+FTC+TAF+EVG |
| 69 | 83 | 730 | 34.2 | <50 | 25 | COB+FTC+TAF+EVG |
| 70 | 44 | 360 | 31.0 | <50 | 35 | RTG+3TC+ABV |
| 71 | 116 | 590 | 23.0 | <50 | 96 | RPV+FTC+TDF |
| 72 | 72 | 930 | 44.8 | 50 | 60 | 3TC+DTG |
| 73 | 106 | 580 | 28.3 | <50 | 34 | BIC+FTC+TAF |
| 74 | 97 | 640 | 29.0 | <50 | 63 | FTC+RPV+TAF |
| 75 | 96 | 540 | 35.0 | 50 | 74 | RPV+FTC+TDF |
| 76 | 121 | 810 | 38.4 | <50 | 45 | BIC+FTC+TAF |
| 77 | 169 | 650 | 35.7 | <50 | 92 | 3TC+ABV+DTG |
| 78 | 163 | 600 | 29.3 | <50 | 70 | FTC+RPV+TDF |
| 79 | 105 | 670 | 32.1 | <50 | 103 | FTC+RPV+TDF |
| 80 | 156 | 700 | 48.5 | <50 | 118 | COB+DRV+DTG |
| 81 | 62 | 1420 | 44.3 | <50 | 54 | 3TC+DTG |
| 82 | 319 | 280 | 27.0 | <50 | 70 | MRV+DRV+RTV+DTG |
| 83 | 100 | 1080 | 37.4 | <50 | 84 | RPV+FTC+TDF |
| 84 | 51 | 860 | 31.2 | <50 | 33 | 3TC+ABV+DTG |
| 85 | 156 | 1240 | 44.0 | <50 | 35 | BIC+FTC+TDF |
| 86 | 158 | 990 | 48.9 | <50 | 81 | 3TC+ABV+DTG |
| 87 | 58 | 600 | 32.1 | <50 | 56 | DTG+3TC |
| 88 | 448 | 1420 | 44.0 | <50 | 42 | COB+DRV+RPV |
| 89 | 31 | 480 | 31.2 | <50 | 28 | DRV+COB+FTC+TAF |

EC, Elite Controller; FTC, emtricitabine; TDF, tenofovir; NVP, nevirapine; ATV, atazanavir; 3TC, lamivudine; EFV, efavirenz; ABV, abacavir; RAL, raltegravir; EVG, elvitegravir; DTG, dolutegravir; DRV, darunavir; RPV, Rilpivirine; TAF, tenofovir alafenamida; BIC, Bictegravir; LPV, Lopinavir; /r, boosted with ritonavir; /c, boosted with cobicistat; UNT, untreated; NA: Not Available.
